# Quackery in the Garb of Piety

**Published:** 1877-12

**Authors:** 


### EXCERPTA.

QUACKERY IN THE GARB OF PIETY.—We have received several letters asking for information about a nostrum which is extensively advertised in a style somewhat similar to that adopted by a quack, " whose sands of life had nearly run out" a number of years ago, but who seems to be at the bottom of the present enterprise, or else has been successfully copied by another. The latter pretends to be a minister of the Gospel and a former missionary at Para, where he asserts to have become acquainted with the wonderful restorative properties of this compound, which consists of extract of corassa apimis eight drachms, extract of selarmo umbelifera four drachms, powdered alkermes latifolia three drachms, and extract of carsadoc herbalis six drachms. In his compassion for unfortunate sufferers, this good and pious quack offers to send this wonderful remedy, securely sealed and accompanied by directions and some excellent advice, for the modest sum of $3.30 by express or $3.50 by mail, which price includes the government stamp, and is just what it costs him; he seeks no other reward than the satisfaction of doing good and the blessing of an approving conscience. His means make him independent, and he goes to all this trouble merely because the drug stores cannot be relied upon to procure new remedies of pure quality.

To inquirers we have simply to state that there are no plants in the Amazon valley, or anywhere else, bearing the above names, and this alone should be sufficient to stamp the enterprise as an imposition, merely undertaken for the purpose of catching the dollars of dupes.

A quack of the same stamp, simulating piety and frankness with the view of the more readily drawing the money of the credulous, was exposed by the *Philadelphia Times* of November 14. His prescription for the sure and speedy cure of consumption, etc., which he offered to put up at $3, was as follows:

Extract Asiatic cannabis sativa, two ounces; extract Asiatic hashish sativa, three ounces; verbena hastata, two drachms; extract diasma, three drachms; pulverized cinchona bark, two ounces; extract cashgar leaves, (blood root) three ounces; inulin, one drachm; loaf sugar, one pound; rum or gin, half pint; cold water, one pint.—*American Journal of Pharmacy.*

